# Gruber’s Reaction in Hog Cholera1Presented at the Fourth Annual Meeting of the United States Experiment Stations Veterinary Medical Association, held at Detroit, Mich., September 4, 1900.

**Published:** 1900-09

**Authors:** R. R. Dinwiddie

**Affiliations:** Arkansas Agricultural Experiment Station, Fayetteville, Ark.


### GRUBER'S REACTION IN HOG CHOLERA.[1]

By R. R. Dinwiddie, M.D., V.S.,
ARKANSAS AGRICULTURAL EXPERIMENT STATION, FAYETTEVILLE, ARK.

In selecting for discussion this subject of serum diagnosis—Gruber's or Widal's reaction—in hog cholera, I have been influenced perhaps less by the desire to communicate my own experiences than to elicit information from the more extensive experience of others. We are all, as investigators in veterinary pathology, necessarily interested in those contagious swine diseases known to the American farmer by the traditional name of "hog cholera," while some of us are now specially engaged, I believe, in their study. My own special studies in this direction are comparatively recent, but I have encountered almost at the outset a difficulty which I think must have been the experience of all engaged in such work, namely, the question of diagnosis between hog cholera properly so called—"swine pest," to use the European name—and "swine plague," and, it may be, other infectious diseases still unstudied.

Our uncertainty is not in all cases removed by the pathological-anatomical findings, nor even by the time-consuming bacteriological research.

Serum diagnosis, which in typhoid fever has been found of sufficient reliability for practical use, seems to offer a promise of aid in the study of these swine diseases. I do not know to what extent it has been used for this purpose, or whether this promise has in practice been fulfilled. I have noticed practically nothing on the matter in European literature, although I confess I have not as yet carefully looked over the reviews for this purpose. Some references to serum diagnosis in swine diseases—the only ones, in fact, which have come under my observation—are contained in the recent reports of the United States Agricultural Department. Dawson, of that department,

[1] Presented at the Fourth Annual Meeting of the United States Experiment Stations Veterinary Medical Association, held at Detroit, Mich., September 4, 1900.

some years ago made a communication in one of the Eastern medical journals of his demonstration of this reaction in the blood of the rabbit inoculated with hog cholera, and in the last annual report of the Bureau of Animal Industry describes the method of applying the test for diagnosis. From other brief references in these reports I infer that this method of diagnosis has been employed in the immunizing experiments in swine diseases carried on by these authors. It has probably been employed also by other Experiment Station workers now present, and from these I hope to hear in discussion something of their experience.

My own studies of this reaction in hog cholera have been almost confined to experimental animals—pigs, rabbits, and guinea-pigs—and have been merely incidental to some immunizing experiments on which I have been engaged.

From the notes of this work the subjoined data referring to the occurrence of this clumping reaction have been extracted:

I ought, first of all, to say something as to the method employed. Nearly all my tests have been made with dried blood. At first the strengths of the dilutions were merely estimated roughly, or rather guessed at; but it was soon found, in my hands at least, impossible to arrive at reliable results without some system by which these dilutions could be more accurately gauged. After some experimenting I finally adopted a method of measuring the volume of the blood samples which has proved entirely satisfactory and has been employed in all the tests referred to in this paper. This method I will demonstrate later if time permits.[1] Dilutions were made with sterile normal salt solution, and the specimens made ready for the microscope by preparing a hanging drop consisting of equal parts—one platinum loop each of the blood solution and a twelve to twenty-four hour bouillon culture. Preparations with a dilution of 1 to 10 as a minimum were first examined, other preparations at 1 to 20, 1 to 40 or higher being then made from each specimen where a reaction was obtained at the lower dilution. I now usually begin with the 1 to 20 dilution, my experience being that nothing is gained by the use of lower dilutions than this. Where a genuine reaction is obtained with a 1 to 10 dilution it is equally plain at 1 to 20, and usually also at 1 to 40. On the other hand, pseudo-reactions

[1] A description of the method referred to has been published in the Journal of Applied Microscopy, vol. iii., No. 6.

are less common at the higher dilution. When for diagnostic purposes one slide only is prepared, a 1 to 20 dilution is perhaps the most reliable, with a time limit of half an hour. In general, however, a second preparation at 1 to 40 will render the results more certain. These statements, I ought to repeat, are based upon an experience with artificially infected animals only, and may not be applicable to the diagnosis of hog cholera on the farms.

*Tests of Normal Blood.* From most of the laboratory animals which I have had occasion to use in the past few months, samples of blood were collected before treatment and tested as to their agglutinating action on hog-cholera bacilli. Out of twenty-six rabbits, with a dilution of 1 to 10, no reaction was obtained in 22. In four pseudo-reactions were present, which, although non-typical, might, if they occurred at a higher dilution, give rise to uncertainty.

No reaction was obtained with the normal blood of the guinea-pig, twelve being examined in all.

In hogs no typical reaction has been found in uninfected cases with a dilution of 1 to 20, but at lower dilutions non-typical reactions may appear, sufficiently marked to give rise to uncertainty. This applies also to the blood of cattle.

*Tests of Blood of Infected Animals.* After the subcutaneous inoculation of hog-cholera bacilli the agglutinating property appears in the blood, at the earliest in four days. It is nearly always present by the sixth day in all animals which I have tested—rabbits, guinea-pigs, pigs, and cattle. Animals which survive the inoculation for the necessary time, as nearly all do, will show this reaction in the great majorityof cases. Nevertheless, there are exceptional instances in which it cannot be demonstrated. I have noticed this in two out of ten guinea-pigs and in one out of sixteen rabbits, with, in addition, one doubtful case. There were no unusual circumstances connected with these cases otherwise.

The two guinea-pigs died ten days after inoculation, and the heart blood was used for the examination in one case, and in the other peritoneal fluid effusion. In the rabbit death occurred four to five days after inoculation, and the heart blood was used for the test.

When non-fatal inoculations are made with cultures of feebler virulence it can be seen that the appearance of the agglutinating property in the blood is occasionally delayed several days beyond the usual time, and this might possibly account for the absence of the reaction in the rabbit.

Out of eleven pigs examined after subcutaneous inoculation with hog cholera cultures all gave a positive result, the reaction appearing in five days. I have found it present also in all of three which were infected by feeding cultures.

*Duration.* In animals which survive inoculation the persistence of the reaction seems to depend upon the severity of the disease induced; hence, upon the virulence of the culture and the size of the dose injected. My observations on this point have been few, but I have noticed in some animals yet on hand that the reaction is still present after four months. On the other hand, the reaction induced by the inoculation of feebly virulent cultures almost disappeared from the blood of pigs after six weeks.

The action of sterilized cultures, whether the sterilization be effected by steam or at a temperature of 60° C., is the same as the living cultures, the reaction appearing about four days after injection Much larger doses are required, and several injections may be needed before this effect is produced. Guinea-pigs and pigs are more resistant, from this point of view, to such injections than rabbits and cattle. The strength of agglutinating power, by repeated injections, may be raised to a high degree, distinct at 1 to 1200 in one of my cases (in the rabbit), but is more transient than after injection of living cultures. Its persistence seems to vary, but may still be present one month after a single injection. It is, according to my experience, more easily produced in rabbits and in cattle than in guinea-pigs and pigs; in fact, I have not succeeded in obtaining the reaction in pigs at all, even with enormous doses of heated cultures.

In tuberculosis and swine plague, in various experimental animals, I have made a few tests, always with negative results.

As to the reaction itself as compared with Widal's reaction in typhoid, typical cases are quite as distinct. The bacillus of hog cholera is smaller than Eberth's bacillus, but individual forms are plainly enough seen with a one-eighth inch dry objective. Clumping, of itself, hardly constitutes a reaction; it must be combined with distinct inhibition of locomotion. Those two phenomena, when the reaction is plain, generally go together. In typical cases there is complete cessation of locomotion—that is, motion across the field, but generally more or less vibratile or whirling movements at the edge of the clumps. In a few cases I have noticed this complete cessation of motion without very distinct clumping. In other cases, where the reaction is feeble, the formation of clumps is the most prominent feature, and motion not distinctly checked. These cases necessarily give rise to doubt as to the genuineness of the reaction, since more or less clumping or grouping of the bacilli often occurs when they are mixed with normal blood or even when untreated. Even after considerable experience some cases will remain in doubt; at least, that is my experience in this disease and in typhoid fever as well.

As to the application of this test to the diagnosis of hog cholera on the farm, I can say very little from personal experience. Since beginning my studies in this direction only two outbreaks of swine disease have been investigated. These were both too far from my laboratory to allow of as thorough investigation as I should have liked, but the post-mortem lesions were what we regard as characteristic of hog cholera. Swine-plague bacilli were isolated in every case, but no hog-cholera bacilli. The sero-diagnostic test, applied to a number of animals in all stages of illness and after death, resulted negatively in every instance. If I were to form an opinion from this limited experience, it would necessarily be either that Gruber's reaction in hog cholera fails us when applied to actual practice, or that our conception of the anatomical lesions characteristic of hog cholera requires revision. Until further experience, however, I prefer not to form any opinion.

